# HDAC8 Inhibitor WK2-16 Therapeutically Targets Lipopolysaccharide-Induced Mouse Model of Neuroinflammation and Microglial Activation

**DOI:** 10.3390/ijms20020410

**Published:** 2019-01-18

**Authors:** Fan-Li Lin, Jing-Lun Yen, Yu-Cheng Kuo, Jaw-Jou Kang, Yu-Wen Cheng, Wei-Jan Huang, George Hsiao

**Affiliations:** 1 School of Pharmaceutical Sciences, National Yang-Ming University, Taipei 112-21, Taiwan; fllin@tmu.edu.tw (F.-L.L.); jjkang@ym.edu.tw (J.-J.K.); 2 Graduate Institute of Medical Sciences and Department of Pharmacology, School of Medicine, College of Medicine, Taipei Medical University, Taipei 110-31, Taiwan; m120102039@tmu.edu.tw (J.-L.Y.); yucheng.kuo@msa.hinet.net (Y.-C.K.); 3 School of Pharmacy, College of Pharmacy, Taipei Medical University, Taipei 110-31, Taiwan; ywcheng@tmu.edu.tw; 4 Graduate Institute of Pharmacognosy, College of Pharmacy, Taipei Medical University, Taipei 110-31, Taiwan

**Keywords:** HDAC8 inhibitor, WK2-16, neuroinflammation, microglia, STAT, Akt

## Abstract

Glial activation and neuroinflammatory processes play important roles in the pathogenesis of brain abscess and neurodegenerative diseases. Activated glial cells can secrete various proinflammatory cytokines and neurotoxic mediators, which contribute to the exacerbation of neuronal cell death. The inhibition of glial activation has been shown to alleviate neurodegenerative conditions. The present study was to investigate the specific HDAC8 inhibitor WK2-16, especially its effects on the neuroinflammatory responses through glial inactivation. WK2-16 significantly reduced the gelatinolytic activity of MMP-9, and expression of COX-2/iNOS proteins in striatal lipopolysaccharide (LPS)-induced neuroinflammation in C57BL/6 mice. The treatment of WK2-16 markedly improved neurobehavioral deficits. Immunofluorescent staining revealed that WK2-16 reduced LPS-stimulated astrogliosis and microglial activation in situ. Consistently, cellular studies revealed that WK2-16 significantly suppressed LPS-induced mouse microglia BV-2 cell proliferation. WK2-16 was proven to concentration-dependently induce the levels of acetylated SMC3 in microglial BV-2 cells. It also reduced the expression of COX-2/iNOS proteins and TNF-α production in LPS-activated microglial BV-2 cells. The signaling studies demonstrated that WK2-16 markedly inhibited LPS-activated STAT-1/-3 and Akt activation, but not NF-κB or MAPK signaling. In summary, the HDAC8 inhibitor WK2-16 exhibited neuroprotective effects through its anti-neuroinflammation and glial inactivation properties, especially in microglia *in vitro* and *in vivo*.

## 1. Introduction

Central nervous system (CNS) injuries and neurological diseases, such as head trauma and neurodegenerative diseases, create enormous individual and financial burdens. Much evidence has revealed that neuroinflammation plays a critical role in the development and progression of these neuropathological conditions [1,2]. Microglial activation, astrogliosis, and the release of inflammatory mediators are the major immune components of neuroinflammation. Microglia are the primary immune glial cells in the CNS and predominantly contribute to neuroinflammation. Microglia are activated and mediate neuroinflammatory responses under pathological conditions via phagocytosis, antigen presentation, and the production of proinflammatory mediators, including interleukin-1 (IL-1) and tumor necrosis factor-α (TNF-α), and oxygen radicals, which results in the propagation of neuronal death and cerebral damage [3,4]. *In vivo* studies of the toll-like receptor 4 (TLR-4) agonist LPS-induced neuroinflammation model revealed that the presence of microglia is necessary in neuroinflammation-mediated toxicity in brain tissue, in which neurons are vulnerable to injury [5]. Astrocyte activation also amplifies the neuroinflammatory responses via the production of neurotoxic factors [6].

The signaling pathways including nuclear transcription factor kappa-B (NF-κB), mitogen-activated protein kinases (MAPKs), and Janus kinasesignal transducers and activators of transcription (JAK-STATs) are involved in microglia-mediated neuroinflammatory responses, such as TNF-α, IL-1β, IL-6, inducible nitric oxide synthase (iNOS) and cyclooxygenase-2 (COX-2) production [7]. LPS or β-amyloid protein exposure activates STAT-3 in microglia. STAT-3 is translocated into the nucleus to induce the expression of various inflammatory genes [8,9]. Previous study revealed that the iNOS promoter region contained NF-κB and STAT binding elements, and specific blockade of NF-κB and STAT activity attenuated neuroinflammatory mediator production in TLR-2/-4 agonist-stimulated microglia [10]. IκB degradation plays a critical role in NF-κB activation, and its degradation is regulated through phosphorylation of IKK by Akt in activated microglia [11]. Suppression of MAPKs and Akt downregulates microglial over-activation and prevents subsequent neuronal death [12,13]. Therefore, the inhibition of microglia dysregulation and subsequent neuroinflammation is increasingly recognized as a strategic target for the treatment of inflammation-mediated neurological diseases.

Previous studies have revealed that epigenetic modifications are critical for transcriptional regulation and gene expression, especially histone acetylation. Inhibition of the epigenetic regulator histone deacetylase (HDAC) regulated the inflammatory response and produced neuroprotective effects in various neurological disorders [14,15]. For example, an *in vivo* study demonstrated that HDAC inhibitors attenuated neuroinflammatory responses, such as the suppression of TNF-α and iNOS in model of cerebral ischemia, and protected against traumatic brain injury from activated microglia-mediated neurotoxicity [14,16]. Pan-HDAC inhibitors such as sodium butyrate, suberoylanilide hydroxamic acid (SAHA), and trichostatin A (TSA) were found to reduce microglia-mediated neuroinflammationin response to LPS *in vivo* and *in vitro* [17,18]. Specific blockade of HDAC8 inhibited the release of proinflammatory cytokines, such as TNF-α and IL-1β, from monocytes in response to LPS *in vitro* and *in vivo* [19]. Our previous study revealed that a novel HDAC8 inhibitor, (*E*)-N-hydroxy-4-methoxy-2-(biphenyl-4-yl)cinnamide (WK2-16), attenuated the production of inflammatory mediators, such as matrix metalloproteinases (MMPs) and IL-6, in monocytic THP-1 cells and decreased serum TNF-α and IL-6 levels in an endotoxemic mouse model [20]. Therefore, HDAC8 inhibitors may be therapeutic in the treatment of neuroinflammation-mediated diseases.

The present study investigated the neuroprotective effects of WK2-16 in a mouse model of intrastriatal LPS administration-induced neuroinflammation. The anti-inflammatory mechanisms were examined using microglial BV2 cells in response to LPS. Our data revealed that WK2-16 significantly prevented the LPS-induced neurological deficits, astrogliosis, and microglia-mediated neuroinflammation *in vivo* and *in vitro*. The cellular studies suggested that anti-inflammatory mechanism of WK2-16 was a restraint of STAT-1/STAT-3 and Akt signaling pathway activation. These results suggest that WK2-16 therapeutically targets glial activation-associated responses, especially microglia, in neuroinflammation-mediated diseases.

## 2. Results

### 2.1. WK2-16 Attenuated the Inflammatory Responses and Improved Neurological Functions in LPS-Induced Neuroinflammation in C57BL6 Mice

**Figure 1:**
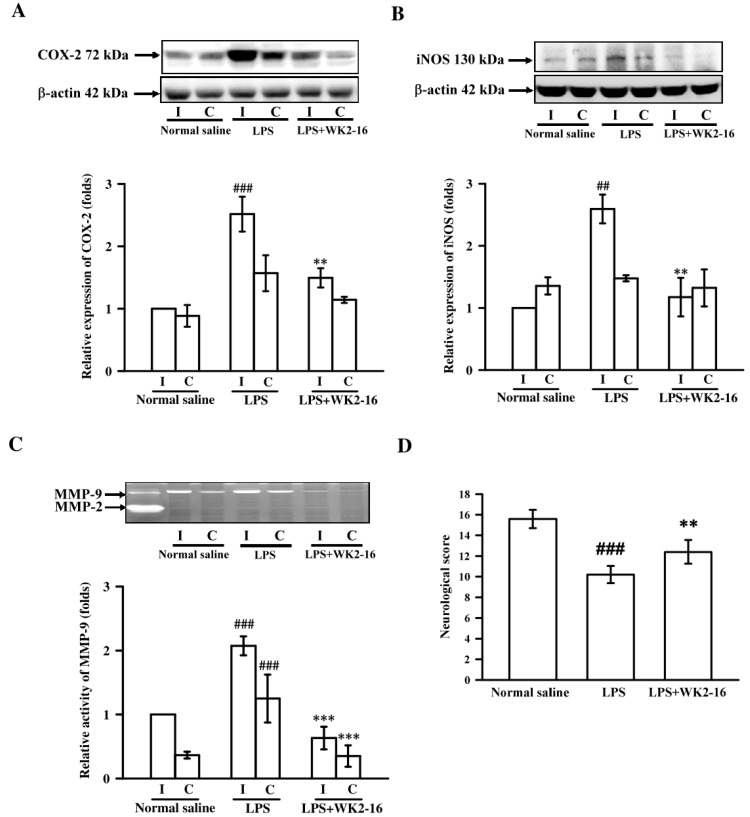
WK2-16 suppressed inflammatory mediator production and protected against functional neurological deficits in LPS-induced neuroinflammation in C57BL6 mice. Protein levels of (**A**) COX-2 and (**B**) iNOS were assessed eight hours after intrastriatal normal saline or LPS injection (4 μg/3 μL) in the presence or absence of WK2-16 (30 mg/kg) using Western blotting. The results are expressed as fold changes normalized to β-actin. (**C**) MMP-9 gelatinolytic activity in brain tissue homogenates was evaluated using zymography. (**D**) Neurological scores according to neurological function changes were recorded eight hours after normal saline or LPS injection in the presence or absence of WK2-16 in C57BL/6 mice. Representative bands shown in Western blots and zymography were classified as the ipsilateral site (LPS-injected brain) and contralateral side (non-operated site). The quantification values are presented as the means ± SD of results from 3–5 animals. I: ipsilateral; C: contralateral. ^##^ *p* < 0.01, ^###^ *p* < 0.001 compared to the normal saline-injected group; ** *p*< 0.01, *** *p*< 0.001 compared to the LPS-injected group treated with vehicle.

We induced neuroinflammation in C57BL6 mice by intrastriatal injection of LPS (4 μg/3 μL) to analyze the anti-neuroinflammatory effects of WK2-16. As compared with the normal saline group, intrastriatal injection with LPS markedly induced COX-2 and iNOS expression by 2.5 ± 0.3- (*p* < 0.001) and 2.6 ± 0.2-fold (*p* = 0.002) in the ipsilateral brain, respectively. Eight hours after intrastriatal LPS stimulation, WK2-16 (30 mg/kg) dramatically attenuated COX-2 and iNOS expression by 1.5 ± 0.2- (*p* = 0.004) and 1.2 ± 0.3-fold (*p* = 0.003) as compared to vehicle-treated group, respectively (Figure 1A,B). Cerebral MMP-9 activity, accessed by zymography, was significantly increased by 2.1 ± 0.1-fold (*p* < 0.001) in LPS-injected group compared to the normal saline-injected group (1.0 ± 0.0-fold). Treatment with WK2-16 significantly attenuated the LPS-increased degradatory activity of MMP-9 in the ipsilateral brain by 0.6 ± 0.2-fold (*p* < 0.001) (Figure 1C).

Modified neurological scores based on motor and sensory functions, including spontaneous activity, symmetry in the movement of four limbs, forepaw outstretching, climbing, body proprioception, and response to vibrissae touch [21], were assessed eight hours post surgery to evaluate neurological damage. The neurological score in the intrastriatally normal saline-injected group was approximately 15.6 ± 0.9 at baseline. A marked reduction of the neurological score was recorded after intrastriatal injection with LPS (10.2 ± 0.8, *p* < 0.001), and subsequent WK2-16 treatment significantly improved the LPS-injured neurological score to 12.4 ± 1.1 (*p* = 0.002) (Figure 1D).

### 2.2. WK2-16 Inhibited Astrogliosis and Microglia Proliferation in LPS-Challenged C57BL6 Mice

**Figure 2:**
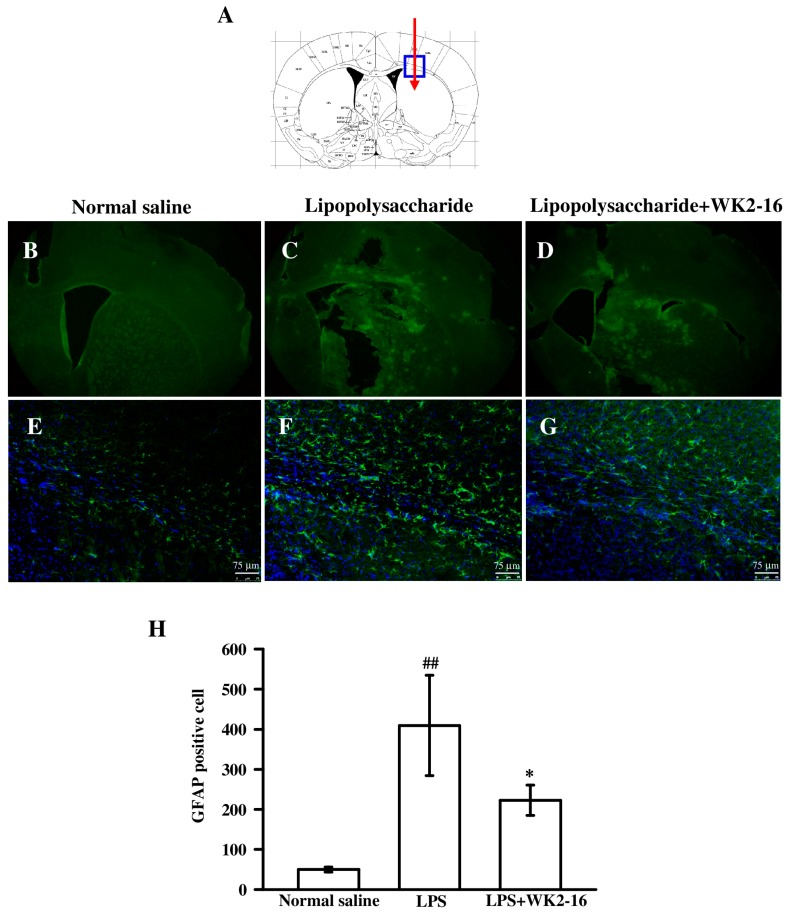
The inhibition of WK2-16 on LPS-induced astrocyte activation in C57BL6 mice brain. Brain sections (50 μm thick) were prepared eight hours after intrastriatal normal saline or LPS (4 μg/3μL) injection in the presence or absence of WK2-16 (30 mg/kg). (**A**) Schematic illustration of the stereotaxic injection site in the caudate/putamen region (red arrow). (**B**–**D**) Representative immunofluorescence staining of GFAP (green) in normal saline- and LPS-injected groups in the presence or absence of WK2-16 (30 mg/kg). (**E**–**G**) Higher magnification fluorescence microscopy of the rectangular area in Figure 2A. Figure 2E,F,G were magnified from Figure 2B, C, and D in the corpus callosum, respectively. GFAP immunostaining was counterstained with DAPI (blue). Scale bar = 75 μm. (**H**) Quantification of cells positive for GFAP. The data are presented as the means ± SD from three animals. ^##^ *p* < 0.01 compared to the normal saline-injected group; * *p* < 0.05 compared to the LPS-injected group treated with vehicle.

**Figure 3:**
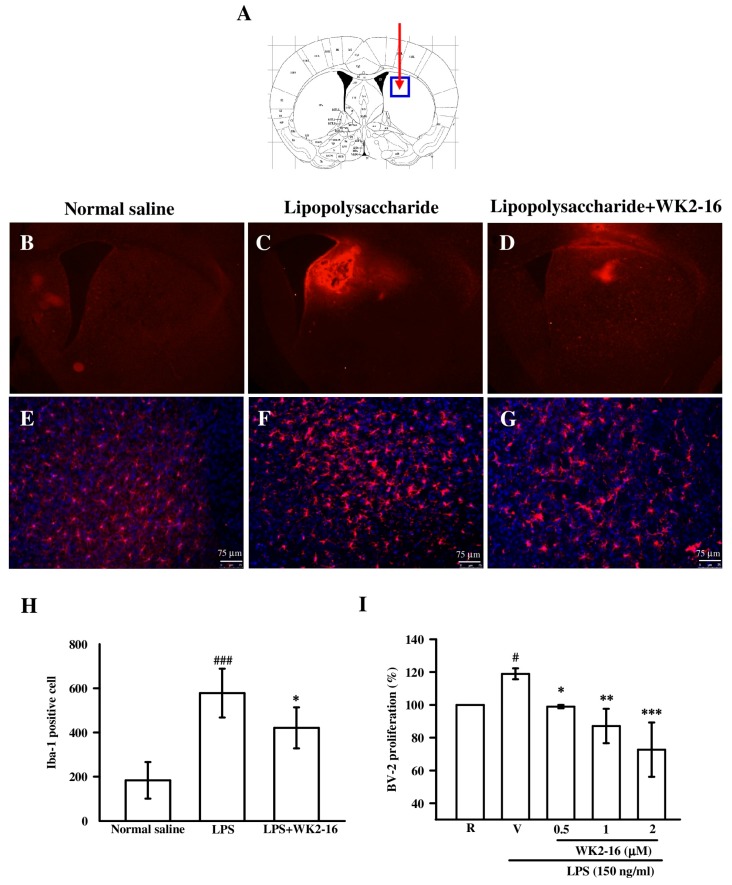
WK2-16 suppressed LPS-induced microglial activation and proliferation. (**A**) Schematic illustration of the site of stereotaxic injection in the caudate/putamen region (red arrow). (**B**,**C**,**D**) Brain sections (50 μm thick) were prepared eight hours after intrastriatal normal saline or LPS (4 μg/3μL) injection in the presence or absence of WK2-16 (30 mg/kg). Microglia were visualized using fluorescent immunostaining with an anti-Iba-1 antibody (red). (**E**,**F**,**G**) Higher magnification fluorescence microscopy of the rectangular area in Figure 3A. Figure 3E,F,G are magnified from Figure 3B, C, and D in the striatum, respectively. Iba-1 immunostaining was counterstained with DAPI (blue). Scale bar = 75 μm. (**H**) Quantification of cells positive for Iba-1. The data are presented as the means ± SD from 4 animals. (I) BV-2 microglia were pretreated with WK2-16 at varying concentrations followed by LPS (150 ng/mL) incubation for 48 h. The relative cell proliferation was determined using the SRB assay *in vitro* (*n* = 3). R: resting; V: vehicle (DMSO). ^#^ *p*< 0.01, ^###^ *p*< 0.001 compared to the normal saline-injected group *in vivo* or resting group *in vitro*; * *p* < 0.05, ** *p* < 0.01, *** *p* < 0.001 compared to the LPS-treated group treated with vehicle in vivo and in vitro.

The activation of glial cells, such as astrocytes and microglia, plays a major role in the pathogenesis of neuroinflammation via mediation of inflammatory responses and alterations in brain-blood barrier (BBB) permeability [2]. Therefore, we examined whether WK2-16 treatment suppressed astrocyte and microglia activation. Brain sections were immunostained with glial fibrillary acidic protein (GFAP) (Figure 2B–D) and ionized calcium-binding adaptor molecule 1 (Iba1) (Figure 3B–D) antibodies, which target astrocytes and microglia, respectively. A significant increase in GFAP-immunoreactive cells in the corpus callosum was found in intrastriatal LPS-injected group compared to the normal saline-injected group (Figure 2E,F). The morphological changes were characterized by enlarged cell bodies and extended processes in GFAP-expressing astrocytes. The increased number and stainability of GFAP immunoreactivity indicated a dramatic astrogliosis (Figure 2F). Subsequent WK2-16 (30 mg/kg) treatment significantly reduced GFAP immunoreactivity compared to the LPS-injected group (Figure 2F,G). The number of cells positive for GFAP was calculated and quantified (Figure 2H). Intrastriatal LPS-injection markedly increased the number of GFAP-positive cell to 409.3 ± 125.3 as compared with the normal saline-injected group (49.7 ± 6.4, *p* = 0.003). Treatment with WK2-16 significantly decreased the number of GFAP-expressing cells (222.7 ± 37.9, *p* = 0.024).

We further examined microglia activation using Iba1. The number of Iba-1 signals in the striatum increased eight hours after intrastriatal LPS administration compared to the normal saline-injected group (Figure 3E,F), which revealed a dramatic reactivation of microglia. Iba-1-immunopositive microglia exhibited a hypertrophic morphology and elongated somas with extended processes (Figure 3F). WK2-16 treatment (30mg/kg) greatly reduced these effects (Figure 3F,G). We further calculated and quantified the number of cells positive for Iba-1 (Figure 3H). LPS-induced neuroinflammation increased the number of Iba-1-expressing cells to 578.0 ± 110.5 compared to the normal saline-injected group (183.5 ± 82.9, *p* < 0.001), and WK2-16 treatment reduced this effect (421.0 ± 92.5, *p* = 0.046). The anti-proliferative effect of WK2-16 in microglial BV-2 cells was assessed using the sulforhodamine B (SRB) assay *in vitro* to examine the inhibitory effect on microglia activation. WK2-16 concentration-dependently (0.5, 1, and 2 μM) inhibited the proliferation of BV-2 cells compared to LPS-stimulated BV-2 cells (*p* = 0.044, *p* = 0.005, and *p* < 0.001, respectively) (Figure 3I). Taken together, WK2-16 treatment markedly suppressed glial activation and proliferation following LPS-induced neuroinflammation.

### 2.3. WK2-16 Inhibited Inflammatory Mediators and Cytokine in LPS-Stimulated Microglial BV2 Cells

**Figure 4:**
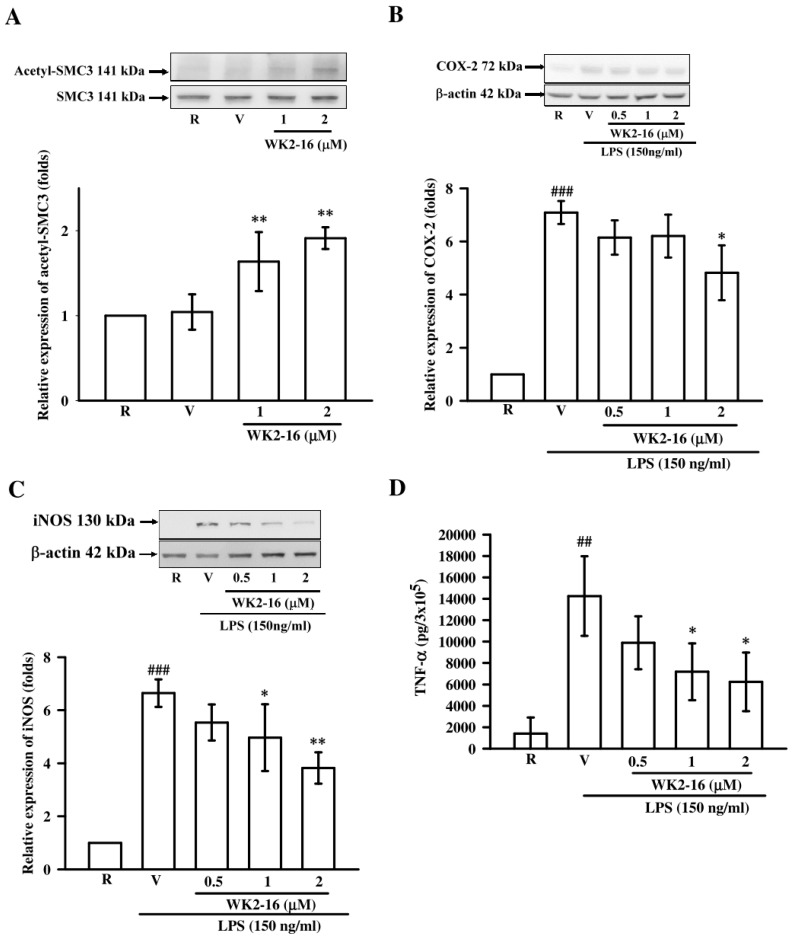
WK2-16 decreased inflammatory enzymes and TNF-α production in LPS-stimulated BV-2 microglial cells. (**A**) BV-2 cells were incubated with WK2-16 for 2 h, and the acetylation level of SMC3 was determined using Western blotting. (**B**,**C**) BV-2 cells were pretreated with vehicle (DMSO) or WK2-16 (0.5, 1, and 2 µM) for 30 min followed by LPS (150 ng/mL) stimulation for 24 h. The expression of COX-2 and iNOS in LPS-stimulated BV-2 cells in the presence or absence of WK2-16 was examined using Western blotting. β-actin was used as the internal control. (**D**) TNF-α levels in conditioned media of cultured BV-2 microglial cells were measured using ELISA. Values are presented as the means ± SD from three independent experiments. R: resting; V: vehicle. ^##^ *p* < 0.01, ^###^ *p* < 0.001 compared to the resting group; * *p* < 0.05, ** *p* < 0.01 compared to the LPS-stimulated group treated with vehicle (DMSO).

Microglia are prime components in maintaining neuroinflammation [22]. Therefore, a mouse microglial BV-2 cell line was used to evaluate the anti-inflammatory effects and mechanisms of WK2-16 under LPS stimulation. The acetylation level of an intracellular target, structural maintenance of chromosomes 3 (SMC3), was evaluated to determine the HDAC8 inhibitory activity of WK2-16 in LPS-stimulated BV-2 cells [23]. WK2-16 significantly enhanced the levels of SMC3 acetylation by 1.6 ± 0.3- (*p* = 0.009) and 1.9 ± 0.1-folds (*p* = 0.003) at 1 μM and 2 μM, respectively, compared to the resting condition (Figure 4A). We further evaluated the anti-inflammatory effects of WK2-16 by measuring inflammatory markers including COX-2, iNOS, and TNF-α in LPS-activated BV-2 microglia [24]. LPS (150 ng/mL) significantly increased the protein expression of COX-2 and iNOS by 7.1 ± 0.4- (*p* < 0.001) and 6.6 ± 0.5-fold (*p* < 0.001) compared to the resting condition, respectively (Figure 4B,C). WK2-16 (2 µM) exerted significant inhibition on COX-2 expression by 4.8 ± 1.0 fold (*p* = 0.010) (Figure 4B). WK2-16 (0.5, 1, and 2 µM) concentration-dependently inhibited iNOS expression by 5.5 ± 0.7-, 4.9 ± 1.3- (*p* = 0.044), and 3.8 ± 0.6-fold (*p* = 0.004), respectively (Figure 4C). The LPS-stimulated TNF-α production in BV-2 cells in the presence or absence of WK2-16 was examined using ELISA. TNF-α level was dramatically increased to 14258.7 ± 3709.5 pg per 3 × 10^5^ cells under LPS stimulation compared to the resting group (1406.3 ± 1507.7 pg per 3 × 10^5^ cells, *p* < 0.001). WK2-16 concentration-dependently downregulated TNF-α production to 9891.3 ± 2476.6, 7182.9 ± 2653.0 (*p* = 0.024), and 6235.9 ± 2739.3 (*p* = 0.020) pg per 3 × 10^5^ cells at 0.5, 1, and 2 μM, respectively (Figure 4D).

### 2.4. WK2-16 Decreased LPS-Stimulated Inflammatory Responses through STAT-1/-3 Signaling Pathway

**Figure 5:**
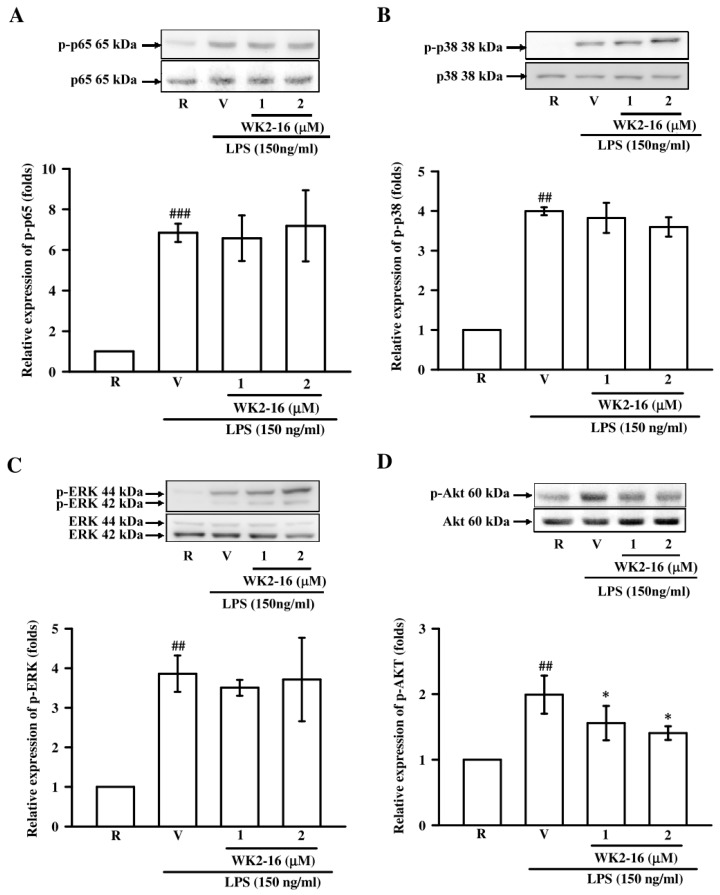
The effects of WK2-16 on LPS-activated pathway signaling in microglial BV-2 cells. Microglial BV-2 cells were pretreated with vehicle (DMSO) or WK2-16 (1 and 2 μM) followed by LPS (150 ng/mL) stimulation for the indicated time. Phosphorylated levels of p65 (A), p38 (B), ERK (C), and Akt (D) in LPS-stimulated BV-2 cells treated with various concentration of WK2-16 were examined using Western blotting. Values are presented as the means ± SD from 3 independent experiments. R: resting; V: vehicle. p-p65: phosphorylated p65; p-p38: phosphorylated p38; p-ERK: phosphorylated ERK; p-AKT: phosphorylated AKT. ^##^ *p* < 0.01, ^###^ *p* < 0.001 compared to the resting group; * *p* < 0.05 compared to the LPS-stimulated group treated with vehicle (DMSO).

**Figure 6:**
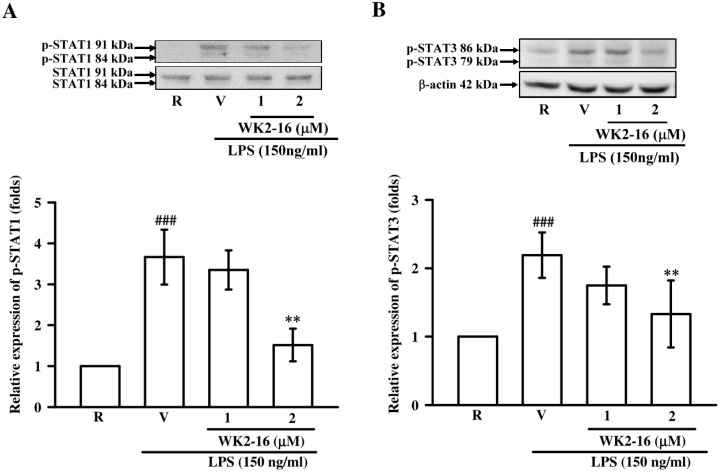
WK2-16 inhibited the activation of STAT-1/-3 in LPS-stimulated microglial BV-2 cells. Microglial BV-2 cells were pretreated with the vehicle (DMSO) or WK2-16 (1 and 2 μM) followed by stimulation with LPS (150 ng/mL) for 3 h. Phosphorylated STAT-1 (*n* = 3) and STAT-3 (*n* = 4) were evaluated using Western blotting. The detection of STAT-1 and β-actin were used as the internal controls. R: resting; V: vehicle; p-STAT1: phosphorylated STAT-1; p-STAT3: phosphorylated STAT-3. ^###^ *p*< 0.001 compared to the resting group; ** *p* < 0.01 compared to the LPS-stimulated group treated with vehicle (DMSO).

We further elucidated the underlying mechanisms of WK2-16 in LPS-stimulated BV-2 microglial cells. First, we evaluated whether WK2-16 (1 and 2 µM) inhibited NF-κB activation under LPS stimulation. LPS challenge dramatically induced p65 phosphorylation (*p* < 0.001), and WK2-16 did not affect this induction (Figure 5A). The effects of WK2-16 on p38 and ERK phosphorylation under LPS stimulation were examined to investigate whether WK2-16 decreased inflammatory responses via MAPK signaling. Exposure to LPS greatly induced p38 (*p* = 0.002) and ERK (*p* = 0.001) phosphorylation, and treatment with WK2-16 did not affect these reactions (Figure 5B,C). We also examined the effect of WK2-16 on Akt activation. Pretreatment with WK2-16 (2 µM) concentration-dependently attenuated the LPS-stimulated phosphorylation of Akt at 1 and 2 µM (*p* = 0.031 and *p* = 0.017, respectively) (Figure 5D). The Janus kinase/signal transducer and activator of transcription (Jak/Stat) signaling has recently identified as a critical regulator of neuroinflammation [25]. We further evaluated the anti-inflammatory effects of WK2-16 (1 and 2 µM) on the STAT signaling in LPS-stimulated BV-2 cells to expand the potential mechanisms. Incubation with LPS significantly increased STAT-1 phosphorylation (*p* < 0.001), and WK2-16 (2 µM) strongly attenuated this activation (*p* = 0.001) (Figure 6A). In addition, the phosphorylation of STAT-3 was induced by LPS (*p* < 0.001), and this effect was attenuated by WK2-16 (2 µM) (*p* = 0.004) (Figure 6B). These results collectively reveal that the anti-inflammatory effects of WK2-16 are regulated through the STAT-1/-3 and Akt signaling pathway.

## 3. Discussion

The present study investigated the application of the HDAC8 inhibitor WK2-16 in neuroinflammation-mediated diseases. The results demonstrated that WK2-16 protected against neurological deficits and reduced neuroinflammatory responses in an intrastriatal LPS-induced neuroinflammation model. The neuroprotection was mediated through the suppression of astrogliosis and microglial activation. Our microglial study revealed that WK2-16 primarily affected STAT-1/-3 and Akt activity to suppress endotoxin-mediated inflammation.

Microglia and astrocytes are innate immune cells that develop and maintain the inflammatory response in the CNS, which is characterized by enhanced proliferation and activation [26]. Extended expression of GFAP is a hallmark for CNS pathologies, and it is primarily distributed in astroglia *in vivo* and *in vitro* [27]. The activation of astroglia produces neuroprotective effect on the retina by enhancing the expression of cytoprotective factors and restoring neurotransmitter balance. However, proliferative astrogliosis can increase vascular permeability and infiltration of toxic compounds, resulting in the acceleration of neurodegeneration [28]. It was also found that proliferative astroglia could release various inflammatory factors during neurodegeneration, leading to myelin damage and axonal loss, especially in corpus callosum. Application of anti-inflammatory and anti-gliosis agent has been shown to effectively reduce demyelination in the corpus callosum [29,30]. WK2-16 significantly reduced GFAP immunoreactivity in the corpus callosum after intrastriatal LPS stimulation in the present study, which demonstrates its anti-astrogliosis activity and the capacity of CNS repairing. In addition, much evidence demonstrated astrocytes as the major source for MMP-9 production in neuropathies. The overproduction of MMP-9 results in disruption of the BBB and death-inducing ligand release, propagating the neuroinflammatory response via the recruitment of immune cells. The inhibition of MMP-9 has been shown to protect against neuronal death and improve neurological functions [31,32]. Therefore, MMP-9 may be a therapeutic target in inflammation-mediated neurological diseases. Our study revealed that WK2-16 dramatically suppressed MMP-9 activation, which may positively correlate with the protective effect of neurological functions. On the other hand, over-expression of the marker Iba1 was primarily found in proliferating microglia/macrophages during the active phase of brain disease. Therefore, suppression of microglial proliferation is sufficient to downregulate microglia-mediated inflammatory functions [33,34]. A previous study reported that activation of PI3K/Akt and STAT-3 signaling increased the survival and proliferation of microglia [35], which revealed that Akt and STAT-3 activation was implied in microglia proliferation. Iba1 immunofluorescence *in vivo* and the microglial SRB assay revealed that WK2-16 significantly attenuated microglia proliferation and reversed morphological changes, including hypertrophic somas and extended processes. The inhibition of proliferation was not involved with cell death because the viability of WK2-16 (2 μM)-treated BV-2 cells was 87.2 ± 4.0% compared to the control cells in the MTT assay (data not shown). Our *in vitro* study further revealed that the possible inhibitory mechanisms of WK2-16 on microglial proliferation in response to LPS may be probable via Akt and STAT-1/-3 inhibition.

The TLR4 activator LPS activates macrophages/microglia and monocytes to release free radicals generated by iNOS in combination with inflammatory mediators and cytokines, including COX-2 and TNF-α, resulting in neuronal damage [36,37]. A previous study indicated that inhibition of mTOR could reduce microglial proliferation and regulate microglial activation via reduction of iNOS and COX-2 expression in response to inflammatory cytokines [38]. NO-mediated toxicity was involved in dopaminergic neuron loss in a model of LPS-induced Parkinson’s disease, and inhibition of iNOS significantly prevented neuronal cell death [39]. In additional, IL-1β- and TNF-α-mediated neuronal death was reported in acute CNS injury and chronic neurodegenerative disease [40]. In the present study, WK2-16 substantially reduced the over-expression of iNOS and COX-2 in intrastriatal LPS-inflamed brains. *In vitro* studies also revealed that LPS-induced microglial iNOS and COX-2 expression and TNF-α production were suppressed by WK2-16. We examined whether WK2-16 affected NF-κB, STATs, and MAPK signaling pathways, which regulate iNOS and COX-2 expression at the transcriptional level, to further clarify the underlying mechanisms. A previous study demonstrated that sufficient activation of STAT-1 was necessary for the full transcription of iNOS in macrophages, whereas NF-κB was activated [41]. STAT-1 and STAT-3 binding sites are widely located in the microglial genome, and manipulation of the constitutive expression of STAT-1 and STAT-3 mimics LPS stimulation, inducing the transcription of hundreds of genes and results in inflammatory cytokines production in microglia [42]. STAT-1/-3 and p300 are found to recruit iNOS and COX-2 gene promoters in LPS-stimulated microglia, and the downregulation of STAT signaling inhibits iNOS and COX-2 gene expression *in vitro* and *in vitro* [43]. In the present study, WK2-16 suppressed the phosphorylation of STAT-1/-3 in LPS-stimulated BV2 microglia, which may positively correlate with the downregulation of iNOS and COX-2 *in vitro* and *in vivo*.

Numerous HDAC inhibitors exhibited promising therapeutic effects in neurological conditions. Pan-HDAC inhibitors, such as TSA, SAHA, valproate, and sodium butyrate, reduced neuroinflammatory responses and exhibited neuroprotective effects in rodent models of stroke, neurodegenerative disease, or acute brain injury [16,44]. TSA and SAHA were found to cross the BBB in clinical trials, which highlights their potent protective effects against Parkinson’s disease [45]. Notably, most studies focused on the use of pan-HDAC inhibitors, which are nonselective HDAC inhibitors. Therefore, it is difficult to clarify which HDACs regulate the inflammatory response and how specific proteins are modified to reduce neuroinflammation [46]. HDAC inhibitors with higher isoform selectivity may reduce the unfavorable adverse effects [47]. A specific HDAC6 inhibitor ameliorated Alzheimer’s disease phenotypes [48], and an HDAC8 inhibition repaired scopolamine-induced learning and memory impairments in animals [49]. The present study addressed whether selective inhibition of Class I HDAC8 by WK2-16 could protect against neurobehavioral deficits and reduce the inflammatory responses in an LPS-induced neuroinflammation mouse model.

Some findings revealed that the inhibition of class I/II HDAC polarized microglia from an inflammatory M1-phenotype toward the protective M2-phenotype with decreased expression of iNOS in a model of traumatic brain injury [50]. The specific inhibition of HDAC1/2 also reduced IL-6 and TNF-α production in LPS-activated microglia [46]. In the present study, WK2-16 downregulated microglial proliferation and morphological changes, positively correlating with the suppression of iNOS expression in LPS-inflamed brains. On the other hand, some HDAC inhibitors affect the interaction between NF-κB and IκBα, the DNA-binding ability, and transcriptional activity of p65, leading to a neuroprotective effect *in vitro* [51,52]. WK2-16 did not reduce p65 phosphorylation in LPS-stimulated microglia, which is consistent with our previous findings in monocytes [20]. The acetylation of histone 3 at lysine 9 by HDACs mediates microglia inflammation in response to LPS. Treatment with the pan-HDAC inhibitor sodium butyrate suppressed proinflammatory (*Tnf-α*, *Nos2*, *Stat1*, *Il6*) and promoted anti-inflammatory (*Il10*) genes via the downregulation of HDAC8 and STAT-1/-3 signaling *in vivo* and *in vitro* [14,53]. According to these studies, the WK2-16-reduced microglial iNOS, COX-2, and TNF-α production could be mediated by HDAC8 and subsequent STAT-1/-3 inhibition. Blockade of STAT-1/-3 signaling has been proven to be neuroprotective in brain injury via an attenuation of inflammatory responses [54,55].

Taken together, the novel HDAC8 inhibitor WK2-16 exerted the neuroprotective effects via suppressing microglial reactivation, astrogliosis, and neuroinflammation. The therapeutic mechanism of WK2-16 is mediated by HDAC8 inhibition and subsequent blockade of STAT-1/-3 signaling, especially in microglia. Further investigation of its mechanism on the biological activity of acetylation is warranted.

## 4. Materials and Methods

### 4.1. Materials

The compound (*E*)-*N*-hydroxy-4-methoxy-2-(biphenyl-4-yl)cinnamide (WK2-16) was provided by Professor Wei-Jan Huang [56]. Dimethyl sulfoxide (DMSO), LPS and SRB were purchased from Sigma-Aldrich (St. Louis, MO, USA). The Iba1 antibody was obtained from Millipore (Temecula, CA). The GFAP antibody was purchased from ProSci Inc. (Poway, CA, USA). 4′,6′-Diamidino-2-phenylindole (DAPI) was obtained from AAT Bioquest, Inc. (Sunnyvale, CA, USA). HIGHDEF^®^ IHC Fluoromount was purchased from Enzo Life Sciences (Farmingdale, NY, USA). β-actin, p-p65, p38, and mouse/rabbit IgG antibodies (DyLight 488) were purchased from GeneTex (Irvine, CA, USA). The acetyl-SMC3 antibody was obtained from MBL international (Woburn, MA, USA). The SMC antibody was purchased from Abcam (Cambridge, MA, USA). The COX-2 and p65 antibodies were purchased from Novus Biologicals (Littleton, CO, USA). The iNOS antibody was purchased from Santa Cruz (Dallas, TX, USA). The p-STAT-1, p-STAT-3, p-Akt, Akt, p-ERK, ERK, p-p38 antibodies were purchased from Cell Signaling (Beverly, MA, USA). The horseradish peroxidase (HRP)-conjugated anti-rabbit/mouse secondary antibodies were purchased from Jackson ImmunoResearch (West Grove, PA, USA).

### 4.2. Animals

Male C57BL/6 mice approximately 25–27 g body weight were purchased from BioLASCO Taiwan Co., Ltd. (Taipei, Taiwan). The animals were maintained in an environment with a 12-h light/dark cycle at 25 ± 1 °C with free access to food and water. The Institutional Animal Care and Use Committee of Taipei Medical University approved all animal use protocols (LAC-2017-0182, 25 July 2017), and animals were operated in accordance with guidelines of ARVO statement for ophthalmic and vision research.

### 4.3. Stereotactic Injections and WK2-16 Administration

C57BL/6 mice were processed for intrastriatal LPS injection as previously described [57]. LPS was dissolved in sterilized normal saline and 3 μL (equivalent to 4 μg LPS) was injected. Control animals were received 3 μL of sterilized normal saline. In brief, the normal saline or LPS solution was given stereotaxically into the caudate/putamen region of the right brain with coordinates of 0.3 mm anterior, 2.0 mm lateral, and 3.8 mm ventral to the bregma. Animals were decapitated eight hours after LPS injection, and brains were quickly harvested.

The dose of WK2-16 (30 mg/kg) was used according to a previous study [20]. This compound was dissolved in a co-solvent (ethanol: cremophor: saline = 1: 1: 8). The treatment group received a single dose of WK2-16 intraperitoneally 30 min after intrastriatal LPS microinjection. The controlled group received intraperitoneally injections with the cosolvent.

### 4.4. Neurological Score

A modified neurological score was used to assess neurological function on a scale of 3–18, in accordance with Garcia et al. [21]. The neurological score was composed of motor and sensory tests, including spontaneous activity, symmetry in the movement of four limbs, forepaw outstretching, climbing, body proprioception, and response to vibrissae touch. Mice were scored for clinical symptoms prior to sacrifice. Mice with lower neurological scores exhibited greater neuroinflammation than mice with higher neurological scores.

### 4.5. Western blot Analysis

Homogenates of brain tissue and microglia cell lysates were prepared as described previously [58]. Samples were subjected to SDS-PAGE and transferred onto nitrocellulose membranes. Each specific primary antibody was immersed with the nitrocellulose membrane at 4 °C overnight. The membrane was incubated with homologous HRP-conjugated secondary antibodies, followed by detection of immunoreactive bandsusing ECL. Densitometry was performed using BioLight software V2000.01 (Uhldingen-Mühlhofen, Germany). The optical density of specific protein was normalized to an internal control and expressed as a ratio to control.

### 4.6. Gelatin Zymography Analysis

MMP-9 activation was evaluated using gelatin zymography as previously described [59]. The degradation of gelatin represents MMP-9 activity. The gelatinolytic zones were acquired using an IP-008-SP Photo-print digital imaging system (Vilber Lourmat, Marue La Vallee, France), and the image was quantified using BioLight software V2000.01 (Uhldingen-Mühlhofen, Germany).

### 4.7. Immunofluorescence

Mice were transcardially perfused with 4% paraformaldehyde (PFA), and brains were resected immediately and fixed in 4% PFA at 4 °C overnight. The brain was dehydrated in 30% sucrose, followed by serial sectioning at a thickness of 50 μm using a cryostat (Leica SM2010R; Nussloch, Germany). The following procedure was executed, as described previously [58]. Brain sections were incubated with anti-GFAP and anti-Iba1 antibodies at 4 °C overnight, followed by incubation with the homologous fluorescence-conjugated secondary antibody DyLight 488 for 2 h at room temperature. Samples were counterstained with DAPI. All fluorescence images were captured using an Eclipse 80i fluorescence microscope (Nikon Instruments, Melville, NY, USA). The fluorescence content of GFAP and Iba-1 was quantified using ImageJ software (Bethesda, Rockville, MD, USA).

### 4.8. Microglial Cell Culture

The mouse BV-2 microglia cell line was cultured in high-glucose Dulbecco’s modified Eagle’s medium (DMEM) supplemented with 10% heat-inactivated fetal bovine serum, penicillin (90 units/mL), streptomycin (90 μg/mL), L-glutamine (3.65 mM), and NaHCO_3_ (23.57 mM) in a humidified 95% O_2_/5% CO_2_ environment at 37°C. The cell culture conditions and cell treatments were previously described [24]. WK2-16 for the *in vitro* studies was dissolved in DMSO. DMSO was used at a constant concentration of 0.1% (vol/vol) in each experiment.

### 4.9. MTT Assay

Colorimetric MTT assays were used to determine cell viability, as described previously [24]. A total of 3 × 10^5^ BV-2 cells were seeded in each well of a 12-well plate and treated with various concentrations of WK2-16 for 22.5 h. MTT was added followed by the further incubation for 1.5 h. Cells were lysed in 1 mL DMSO, and the absorbance values of medium were measured using a microplate reader at 550 nm (Thermo Multiskan GO, Ratastie, Finland).

### 4.10. Sulforhodamine B (SRB) Assay

The cell proliferation of microglia BV-2 cells was determined using the SRB assay, as described previously [60]. BV-2 cells (5 × 10^3^) were dispensed in a 96-well plate and pretreated with varying concentrations of WK2-16 (0.5, 1, and 2 μM) for 30 min. Cells were subsequently incubated with LPS (150 ng/mL) for an additional 48 h. Cells were fixed in 10% trichloroacetic acid for 10 min and washed with distilled water twice to remove excess trichloroacetic acid. Cells were stained with a 0.4% SRB solution dissolved in 1% acetic acid for 10 min and washed with 1% acetic acid twice to remove excess dye. The protein-bound dye was dissolved in 100 μL Tris-based solution (10 mM), and the absorbance was measured at 515 nm using a microplate reader. Relative cell proliferation was calculated as the absorbance of the treated group/the absorbance of the resting group.

### 4.11. Enzyme-Linked Immunosorbent Assay (ELISA)

TNF-α levels in the conditioned media of BV-2 microglial cells were detected using an ELISA kit according to the manufacturer’s instructions (BioLegend, San Diego, CA, USA).

### 4.12. Statistical Analyses

All data are expressed as the means ± SD. Statistical analyses were performed using one-way analysis of variance in Sigma Stat v3.5 software. Statistical differences between experimental groups were determined using the Student-Newman-Keuls test. A *p*-value < 0.05 was considered statistically significant.
